# Ultrasensitive detection of Staphylococcal enterotoxin B in milk based on target-triggered assembly of the flower like nucleic acid nanostructure†

**DOI:** 10.1039/c9ra08869e

**Published:** 2019-12-20

**Authors:** Xiaohui Xiong, Yun Luo, Yichen Lu, Xiong Xiong, Yi Li, Yuanjian Liu, Lixia Lu

**Affiliations:** Coll. Food Sci. & Light Ind., Nanjing Tech University Nanjing 211816 China lucias_cumt@163.com llxhn66@126.com +86-25-58139527 +86-25-58139432

## Abstract

A rapid and ultrasensitive method is described for the detection of Staphylococcal enterotoxin B (SEB). It is based on the formation of the flower like nucleic acid nanostructure by integrating (a) target-induced triggering of DNA release with (b) signal amplification by a hybridization chain reaction (HCR). Firstly, partially complementary pairing of aptamer and trigger DNA forms a duplex structure. The capture DNA (cpDNA) is then placed on the surface of gold electrode through gold-thiol chemistry. In the presence of SEB, the aptamer-target conjugate is compelled to form. This causes the release of trigger DNA owing to a strong competition between aptamer and SEB. Then, the trigger DNA is subsequently hybridized with the partial complementary sequences of the cpDNA to trigger HCR with three auxiliary DNA sequences (referred to as MB1, MB2, MB3). Finally, the flower like nucleic acid nanostructures are formed and allow numerous hexaammineruthenium(iii) chloride ([Ru(NH_3_)_6_]^3+^, RuHex) to be absorbed on the DNA by electrostatic interaction, and thus amplify electrochemical signal. Under optimal conditions, the chronocoulometry charge difference increases linearly with the logarithm of the SEB concentrations in the range from 5 pg mL^−1^ to 100 ng mL^−1^ with a detection limit as low as 3 pg mL^−1^ (S/N = 3).

## Abstract 2

A rapid and ultrasensitive method is described for the detection of Staphylococcal enterotoxin B (SEB).

## Graphical abstract

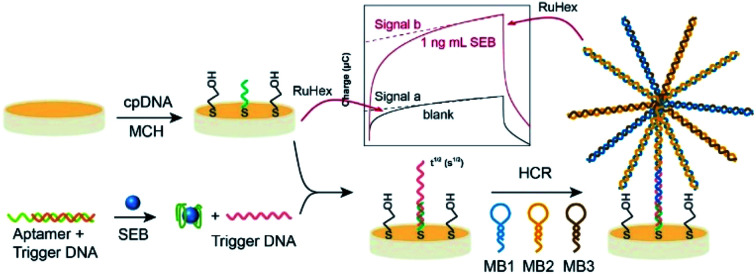

## 1. Introduction

As one of the most common food borne pathogens, Staphylococcal food poisoning is considered as one of the most prevalent causative agents of food-borne illness associated with human health and food safety worldwide. The enterotoxin is produced mainly by *Staphylococcus aureus* and *Escherichia coli*. The food poisoning is the resulting of Staphylococcal enterotoxins (SEs), which includes SEA, SEB, SEC1/2/3, SED, SEE and SEG–SEV, but except for SER.^1^ Because SEs has stable nature throughout food process, which can still cause illness with human being after the *Staphylococcus aureus* inactivated. Notably, SEB, as a superantigen, even though at a very low level can cause death with human.^2^

Conventional identification assays for SEB detection have animal experiment, surface-enhanced Raman scattering (SERS),^3^ polymerase chain reaction (PCR),^4^ enzyme-linked immunosorbent assay (ELISA),^5^ magnetoelastic-sensing,^6^ liquid chromatography mass spectrometry^7^ as well as photonic crystal lab-on-a-chip^8^ and so on. There are some limitations with these methods, such as requirement of expertise personnel for data interpretation, complicated procedures specific equipment, lengthy protocol times, and expensive. Most of these assays depend either directly or indirectly on antibodies against target agent as well, which are sensitive to temperature, pH alterations and finite lifetime.^9^ Consequently, it is particularly critical to improve and perfect the detection technology of enterotoxin in the field of food safety detection.

In comparison with these methods, electrochemical sensor is powerful analytical instrument due to its portability, self-contained, sensitive and low-cost. It is a highly sensitive and selective response tool rapidly, which advanced functionalized nanomaterials based electroanalytical strategies.^10^ The modified electrode surface which affects the sensitivity of biosensors and bioactivity of biomolecules is the most significant step. To obtain a better performance, gold nanomaterial has been introduced to improve the sensitivity of electrochemical sensors due to its special electronic and catalytic properties.^11^ Zhang developed a sensitive electrochemical detection of human methyltransferase based on a dual signal amplification strategy coupling gold nanoparticle–DNA complexes with Ru(iii) redox recycling and a detection limit down to 0.3 U mL^−1^ for 8 MCF-7 cells.^12^ Yang *et al.* reported a strategy through the disassembly of triple-helix stem structure to accelerate the transduction of signal probes, achieving a significant amplified fluorescence resonance energy transfer (FRET) signal readout with the limit of detection of 0.23 pg mL^−1^.^13^ Wu proposed a sensitive aptamer-based fluorescence method for ochratoxin, which allowed for a low limit detection of 0.08 ng mL^−1^.^14^ Song reported a triply amplified electrochemical assay based on a catalytic hairpin assembly with a DNAzyme and any analytical range that covers the 100 pM to 5 μM Pb(ii) concentration range.^15^ These experiments mean that the electrochemical method has the promising applications for the on-site analysis.

Nucleic acid aptamers, which are single-stranded oligonucleotides (usually shorter than 80 nucleotides) are typically selected from random sequences of nucleic acid database by an *in vitro* evolution process, termed systematic evolution of ligands by exponential enrichment or systematic evolution of ligands by exponential enrichment (SELEX).^7^ Aptamers showed the ability to bind to a vast range of molecular targets with high specificity and affinity, which makes them used for detecting SEB.^16^ The combination of multiple nucleic acid aptamers can not only improve the affinity of specific binding target molecules, but also reduce the cost. Therefore, aptamers-based electrochemical technology has a potential application as bio-probes for bio-sensing application to detect due to its low cost, more stable, easier modification, easier synthesis, and higher affinity.^9^ The nucleic acid amplification technologies, as hybridization chain reaction (HCR), loop-mediated isothermal amplification (LAMP), rolling circle amplification (RCA) and catalytic hairpin assembly (CHA) have been widely used. In order to obtain great reliability to improve the low detection limit and the sensitivity of target detection, an amplifiable nucleic acid circuit, HCR is usually applied in this paper. Yang presented a cascade nucleic acid amplification strategy for fluorometric aptamer based determination of model protein carcinoembryonic antigen (CEA), which has high sensitivity and a linear range that covers the 1 pg mL^−1^ to 2 ng mL^−1^ CEA concentration range, with a 0.3 pg mL^−1^ detection limit.^17^ Zou has demonstrated a novel electrochemiluminescence (ECL) method for histone acetyltransferases activity analysis by integrating HCR signal amplification and ECL silver clusters.^18^ The analytical platform allowed the sensing of the histone acetyltransferases with a detection limit of 0.49 nM. Ren showed a four-way branch migration HCR strategy for signal amplification on the FRET biosensor, which might show great potential in food safety and clinical diagnosis.^19^

**Fig. 1:**
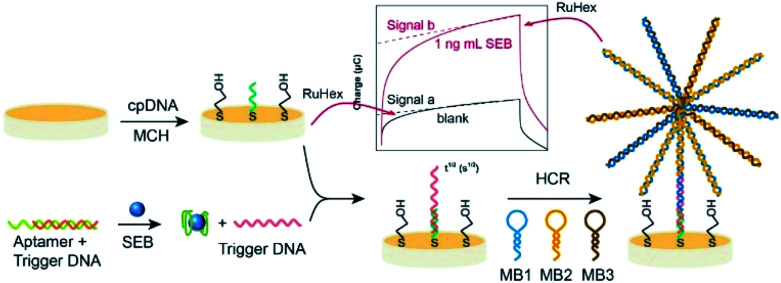
The schematic diagram of the SEB sensing based on the flower like nucleic acid nanostructure assembled on gold electrode.

In this paper, an electrochemical assay was described which employed target-triggered assembly of the flower like nucleic acid nanostructure on a gold electrode to detect SEB. The fabrication process of the flower like nucleic acid nanostructure is illustrated in Fig. 1. Under optimal conditions, the capture DNA (cpDNA) chain is fixed on the electrode surface, and the MCH was used to seal the site. The electrical signal is measured through the electrochemical workstation. Signal a as the background by adding 1 ng mL^−1^ SEB to trigger DNA and then signal b shown that nucleic acid chain reaction was activated to form flower-like nucleic acid nanostructure. The capture DNA (cpDNA) chain fixed on the electrode will selectively hybridize with the trigger DNA. When introducing other three special auxiliary DNA sequences (referred to MB1, MB2, MB3), the DNA sequences with different stem ring structures were combined by the base complementary pairing principle, it means that HCR was activated to form flower-like nucleic acid nanostructure. The number of flower-like nanostructures is proportional to the amount of SEB. The SEB is qualitatively and quantitatively analyzed by detecting the electrical signals of flower-like nucleic acid nanostructures. To amplify the electrochemical signal, hexaammineruthenium(iii) chloride ([Ru(NH_3_)_6_]^3+^, RuHex) was used as an electrochemical probe, each of electrode can generate an electrochemical signal at suitable potential by electrostatic reaction absorbing on dsDNA. The applicability and reliability of the method was demonstrated by analyzing (spiked) milk samples, and the results were compared to those obtained with an ELISA kit, which gives a potential application prospect in SEB detection.

## 2. Experimental

### 2.1 Materials and instrumentation

Staphylococcal enterotoxins (SEA, SEB, and SEC) were obtained from Shanghai Jianglai Industrial Limited By Share Ltd. (Shanghai, China). Aflatoxin B1 (AFB1) and Ochratoxin A (OTA) were offered by Academy of State Administration of Grain (Beijing, China). Chloramphenicol (CAP), Bull serum albumin (BSA) and Casein (CS) were ordered by WITEGA Laboratorien Berlin-Adlershof GmbH (Berlin, Germany). Tris-(2-carboxyethyl) phosphine hydrochloride (TCEP) was supplied by Shanghai Macklin Biochemical Co. Ltd. (Shanghai, China). 6-Mercapto-1-hexanol (MCH) and RuHex were received from Sigma-Aldrich Co. Ltd. (St. Louis, MO). A SEB ELISA kit was provided by YaJi Biotechnology Co., Ltd. (Shanghai, China). All of other analytical grade reagents were used as bought directly without further purification. Ultrapure water (18.2 MΩ cm^−1^ at 25 °C) from a Pure Water system (GWA-UN1-F40, Persee, China) was used in all experiments. All DNA oligonucleotides were synthesized by Shanghai Sangon Biological Engineering Technology & Services Co. Ltd. (Shanghai, China) and purified using high performance liquid chromatography (HPLC). Table S1† was shown the synthetic sequences.

All electrochemical measurements were measured on a CHI 760D electrochemical analyzer (Chenhua Instruments, Shanghai, China). A traditional three-electrode system, which includes a 2 mm-diameter modified gold working electrode, Ag/AgCl reference electrode and a platinum wire as the auxiliary electrode was used. In this experiment, the chronocoulometry (CC) was used in 10 mM Tris–HCl buffer (pH 8.0) with the following parameters, the range of point from −500 mV to 0 mV, potential step from −0.6 V to 0.2 V, scan time = 500 ms.

### 2.2 Assembly of the flower like nucleic acid nanostructure

Before modify, polished the electrode on a microcloth for a mirror surface with 0.05 μm α-Al_2_O_3_ powders, after that in the way of sonication with ultrapure water and acetone for 5 min to remove residual impurity. Subsequently, after immersing in a prepared piranha solution for 15 min the gold electrode was drastically rinsed with ultrapure water. Then, a reproducible cyclic voltammetry (CV) characteristic for the gold electrode clean surface was detected by CV in the potential range of −0.6 V to +0.2 V at a scan rate of 0.05 V s^−1^ in a fresh-obtained 0.5 M H_2_SO_4_ solution. After washing with amount of ultrapure water and drying in nitrogen, a mixture of 10 μL thiolated cpDNA (1 μM) and TECP (1 mM) was cast on the gold electrode overnight at room temperature and then washed by Tris–HCl buffer (10 mM Tris–HCl, pH 8.0) three times to remove the unbound cpDNA. Subsequently, the resulting electrode was then incubated with MCH solution for 1 h at room temperature followed by rinsing with Tris–HCl buffer. Meanwhile, 2 μL SEB aptamer (1 μM) and 5 μL trigger DNA were combined by the principle of base complementary pairing at room temperature for 2 h. In the presence of SEB, the aptamer-target conjugate was compelled to form. This causes the release of trigger DNA owing to a strong competition between aptamer and SEB. Then, the trigger DNA is subsequently hybridized with the partial complementary sequences of the cpDNA. After a washing step with Tris–HCl buffer, 5 μL MB1 (10 μM), 5 μL MB2 (10 μM) and 5 μL MB3 (10 μM) were mixed to drop onto the electrode surface and incubated at room temperature for 2 h. Finally, after washing three times, the modified electrode was subsequently immersed in Tris–HCl buffer containing 50 μM RuHex. Thus, the gold electrode was successfully assembled with the flower like nucleic acid nanostructure.

### 2.3 Electrochemical measurements

For detection, the aptamer-target conjugate was incubated with different concentrations of SEB for a certain time at room temperature. Then, the above solution was added on cpDNA modified gold electrode to trigger HCR with three auxiliary DNA sequences. Finally, after washing three times, the modified electrode was subsequently immersed in Tris–HCl buffer containing 50 μM RuHex. After that, CC was carried out in 10 mM Tris–HCl buffer (pH 8.0). To assess the specificity and sensitivity, the developed electrodes were tested by analogs or other common toxins of various concentrations.

### 2.4 Sample preparation

In order to evaluate the application capability of the designed electrochemical sensor in the real samples, skim milk and milk powder as the actual sample were performed, which were purchased from the local market in Nanjing, China. The steps for sample pretreatment were according to the China Food Safety Standard (GB 2761-2017): first of all, a mixture of 25 g milk samples and 125 mL Tris–HCl buffer were centrifuged at 3500 rpm for 10 min. After that, the milk samples and the Tris–HCl buffer at 1 : 19 v/v proportion blended. Subsequently, two phases were observed from bottom to top: the fat layer and the supernatant (100 μL). The obtained supernatant (100 μL) was used for the next electrochemical detection. Finally, known quantities of 1000 ng mL^−1^ SEB standard solution was used to prepare SEB spiked milk samples at different concentrations (0.1, 1, 10 and 25 ng mL^−1^).

### 2.5 Polyacrylamide gel electrophoresis^20^

At the Model DYCP-31E electrophoretic device (Wo De Life Sciences Instrument Co. Ltd., Beijing, China), different measured solution with 2 μL of 6× loading buffer was added into 2% gel electrophoresis in the gel electrophoresis method of the oligonucleotide hybridizations. The obtained gels were stained with GelRed for 20 min with 3 μL, all the mixtures were run at 110 V for 40 min with 0.5× TBE buffer. Then, the Tanon 1000 gel image analysis system (Shanghai, China) was used to scan the gel images.

## 3. Result and discussion

### 3.1 Characterization

**Fig. 2:**
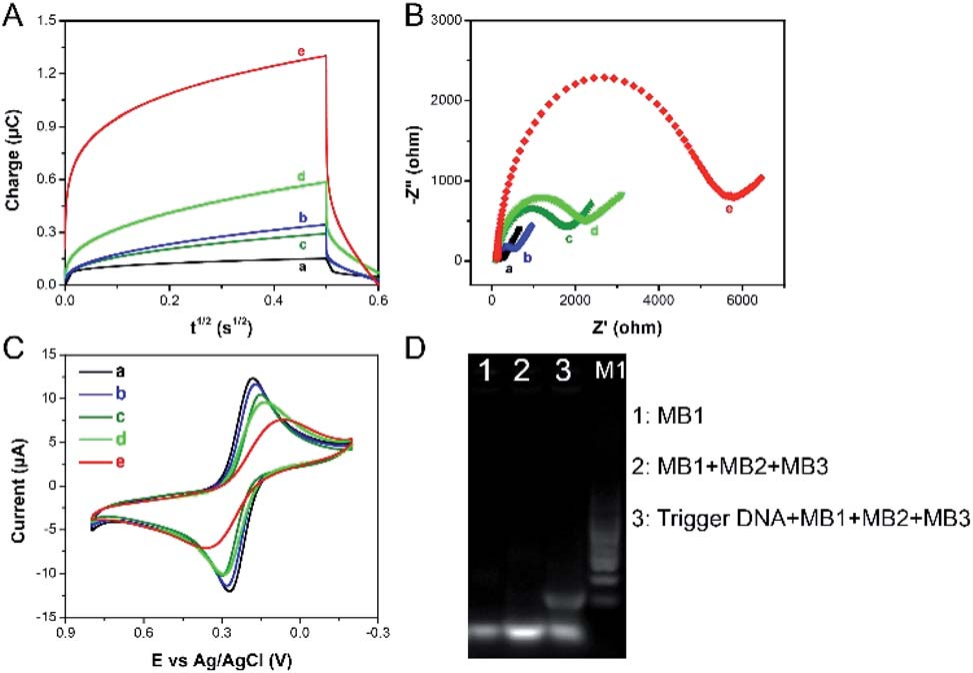
CC (A), EIS (B) and CV (C) assays were used to validate principle experiment. (a) bare gold electrode, (b) cpDNA/gold electrode, (c) MCH/cpDNA/gold electrode, (d) trigger DNA/MCH/cpDNA/gold electrode, (e) HCR/trigger DNA/MCH/cpDNA/gold electrode. Chronocoulometric measurements were performed in 10 mM pH 8.0 Tris buffer containing 50 μM of RuHex. EIS measurements were performed in 1 mM K_3_[Fe(CN)_6_]/K_4_[Fe(CN)_6_] (1 : 1), 100 mM KCl. 1 ng mL^−1^ SEB was used. Polyacrylamide gel electrophoresis evidence (D) for the assembly of DNA nanostructure. Lane 1: 10 μM of MB1; Lane 2: 10 μM of MB1 + 10 μM of MB2+ 10 μM of MB3; Lane 3: 100 nM of trigger DNA + 10 μM of MB1 + 10 μM of MB2 + 10 μM of MB3.

The chronocoulometric technique was used to characterize the formation and function of the designed electrochemical sensor. The curves of charge (*Q*) relative to time (*t*) for the modified electrode are shown in Fig. 2A. Because of negligible nonspecific adsorption of RuHex at the electrode surface, the minimum number of charge is showed in the bare electrode (Fig. 2A, a). The immobilization of the cpDNA on the surface of the modified electrode induces to increase the amount of charge (Fig. 2A, b) by electrostatic interactions with negatively charged phosphate groups and RuHex, which demonstrates that the immobilization of cpDNA on the electrode surface is successful. As expected, when the MCH is blocked, the intensity of electricity changes can be ignored (Fig. 2A, c). In the presence of SEB, the aptamer-target conjugate is compelled to form. This causes the release of trigger DNA owing to a strong competition with SEB. Then, the trigger DNA is subsequently hybridized with the partial complementary sequences of the cpDNA. The electrochemical signal becomes further enhanced (Fig. 2A, d). After that, the HCR was triggered with three auxiliary DNA sequences (referred to as MB1, MB2, MB3) to form the flower like nucleic acid nanostructure. The flower like nucleic acid nanostructure allowed numerous RuHex to be absorbed on the DNA by electrostatic interaction, and signal intensity response was drastically amplified (Fig. 2A, e). The assemble process was also demonstrated by EIS^21^ and CV. Electron transfer impedance (*R*_et_) values (Fig. 2B, a–d) increase stepwise and current signals (Fig. 2C, a–d) decrease stepwise, because the negative charges of DNA prevent repelling electrons from approaching the electrode surface. After HCR is triggered on the electrode, a large increasing *R*_et_ and a small current intensity are observed (Fig. 2B and C, e), suggesting the formation of the flower like nucleic acid nanostructure further inhibited the interfacial electron transfer. On the basis of these results, we can conclude that the formation of the flower like nucleic acid nanostructure is successfully achieved on the gold electrode surface. As shown in Fig. 2D, PAGE was used to demonstrate the formation of the flower like nucleic acid nanostructure. Lane 1 shows the band of 10 μM MB1. No mobility of basic units is observed with the presence of MB1, MB2 and MB3. Then, upon the introduction of trigger DNA, much less mobility is observed due to the selfassembly of building units into fabricated the flower like nucleic acid nanostructure with collective high molecular weight (Lane 3). On the basis of these results, we can conclude that the formation of the flower like nucleic acid nanostructure is successfully achieved on the gold electrode surface.

### 3.2 Optimization of method

**Fig. 3:**
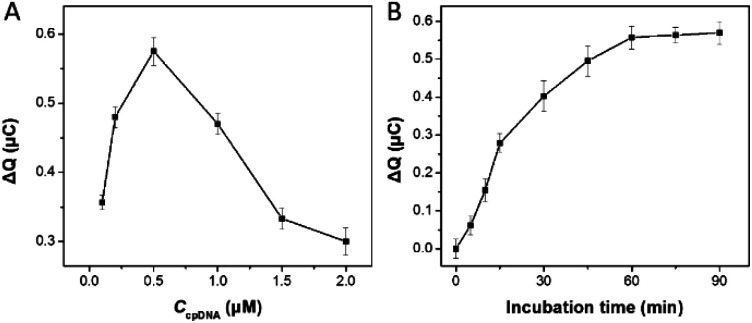
(A) The cpDNA concentration of electrode modification was optimized. 1 ng mL^−1^ SEB was used. (B) Incubation time of biosensor and SEB. Δ*Q* was defined as the difference of redox charge without SEB in SEB and HCR amplification treated with RuHex (Δ*Q* = *Q*_with SEB_ − *Q*_without SEB_).

To obtain the best analytical performance of the designed electrochemical sensor, several experimental conditions influencing the HCR procedure were optimized in this assay. The change in the redox charge of RuHex (Δ*Q* = *Q*_with SEB_ − *Q*_without SEB_) is used to characterize the signal gain. Fig. 3A showed the effect of the cpDNA concentration on the signal response, treated with SEB and without SEB in the present of HCR amplification. Δ*Q* values first increased rapidly to a maximum level at 0.5 μM of cpDNA concentrations and then decreased steeply with increasing the cpDNA concentration. This phenomenon could be explained that excessive density of immobilization of the cpDNA on the electrode surface reduced hybridization efficiency of double-strand DNA. Thus, 0.5 μM of cpDNA concentration was chosen for the following sensing process.

The incubation time of aptamer-trigger DNA conjugate and SEB was investigated. As shown in Fig. 3B, the relative signal response increased rapidly as the incubation time increased and then reached a nearly plateau value at 60 min. Hence, 60 min was considered as the optimal time for the next experiments.

### 3.3 Determination of SEB

**Fig. 4:**
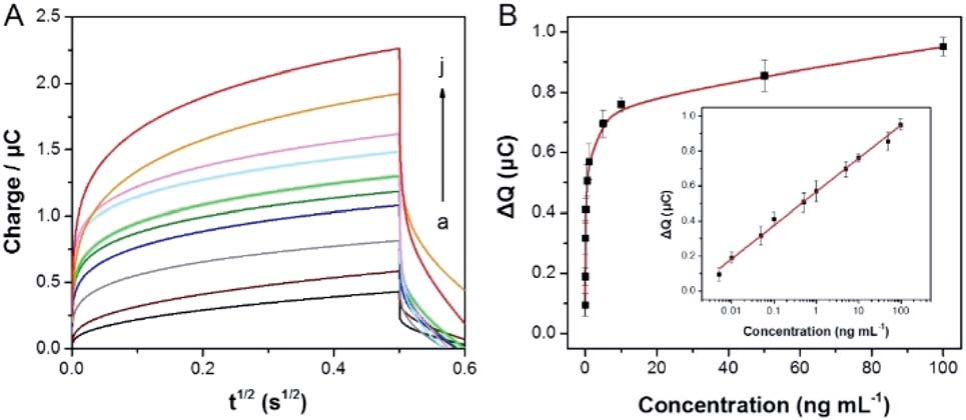
(A) Different concentrations of SEB (from a to j: 0.005 to 100 ng mL^−1^) was measured by chronocoulometry curves. (B) The dependence of the concentration of SEB on Δ*Q*. The inset shows calibration curve corresponding to Δ*Q* as a function of the logarithm concentration of SEB. Error bars showed the standard deviation of three experiments.

In the case of the optimal experimental, the signal of flower like nucleic acid nanostructure was amplified by HCR to detect SEB. As shown in Fig. 4A, the signal response is increasing distinctly, which with increasing the concentration of SEB (0.005, 0.01, 0.05, 0.1, 0.5, 1, 5, 10, 50 and 100 ng mL^−1^). Within the range of 0.005 to 100 ng mL^−1^ (Fig. 4B), it is a good linear relationship between the Δ*Q* values and the logarithm of the concentrations of SEB. Besides, the linear regression equation for the inset is as follows: *y* = 0.5666 + 0.191*x*, which *y* is equal to the Δ*Q* values (μC) and *x* is equal to the logarithm of SEB concentration (ng mL^−1^), in addition, the detection limit (LOD) of 3 pg mL^−1^ was calculated following IUPAC recommendations (3Sb/*b*, where Sb is the standard deviation (*n* = 5) of the blanks, and *b* is the slope value of the respective calibration graph). Furthermore, a comparison of linear ranges and detection limits of other methods and the previously reported methods for SEB is also shown in Table 1. This method is better and more comparability prior to most previous reports.

**Table 1:** Comparison between different sensors for SEB detection

Method	System	Detection range	Detection limit	Ref.
Fluorescence	Carbon dot-DNA and acriflavine nano-assembly	0.1–50 ng mL^−1^	5 ng mL^−1^	22
PCR	Combining BCA with real-time PCR	0.001–100 ng mL^−1^	0.269 pg mL^−1^	23
Colorimetry	Peroxidase-like activity of eggshell membrane-templated gold nanoclusters	0.4–20 ng mL^−1^	0.12 ng mL^−1^	24
Amperometry	Antibody microarray	0–100 pg mL^−1^	5 pg mL^−1^	25
ELISA	A surface functionalized silica matrix	10–0.001 μg mL^−1^	5 ng mL^−1^	26
ELISA	A gold nanoparticle-based enhanced chemiluminescence immunosensor	0.01–50 ng mL^−1^	0.01 ng mL^−1^	27
CC	The flower like nucleic acid nanostructure	0.005–100 ng mL^−1^	3 pg mL^−1^	This work

### 3.4 Selectivity of the method

**Fig. 5:**
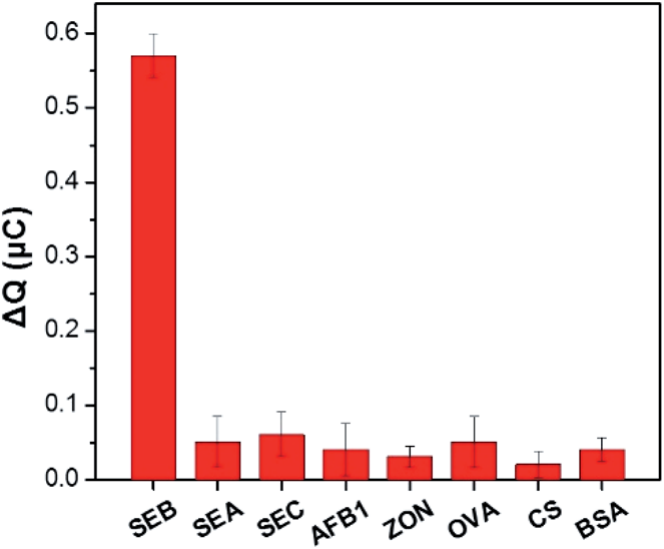
With the presence of SEB, SEA, SEC, AFB1, ZON, OVA, CS and BSA (SEB: 1 ng mL^−1^, SEA: 10 ng mL^−1^, SEC: 10 ng mL^−1^, and 50 ng mL^−1^ for others), the selectivity of the sensing system was measured. Error bars showed the standard deviation of three experiments.

Selective research is important to assess the reliability of a newly developed analytical technique. The study investigated the selectivity of the sensing system was measured in the presence of various SEs (SEB: 1 ng mL^−1^, SEA: 10 ng mL^−1^, SEC: 10 ng mL^−1^) and other common toxins (AFB1, ZON, OVA, CS and BSA: 50 ng mL^−1^). The CC response for the corresponding analytes obtained were displayed and compared in Fig. 5. Only the target SEB to induce a large signal change. On the contrary, the response of the analogs or other common toxins can be ignored. It is attributed to the strong affinity between the aptamer and SEB. These results indicated that the designed electrochemical sensor has remarkable selectivity to SEB.

### 3.5 Determination of SEB in real samples

In order to further demonstrate whether the method was feasible and economic in real bio-environments, we mixed known fixed concentrations of SEB (0.1, 1, 10, 25 ng mL^−1^) for recovery study with that the designed electrochemical sensor in the skim milk and milk power samples, respectively. As shown in Table 2, real sample is no SEB originally based on the calibration plot. Compared obtained results with a commercially available ELISA method, the recovery had no obvious difference in between. What's more, the LOD of ELISA kit is 0.5 ng mL^−1^, the ELISA kit can detect nothing when addition of SEB is 0.1 ng mL^−1^, but the biosensor can. Therefore, the developed method is more sensitive than ELISA, which has a broader market prospects for SEB determination in real samples.

**Table 2:** The applicability of sensor and the ELISA kit to verify through recoveries of SEB in food samples

Sample	Original (ng mL^−1^)	Added (ng mL^−1^)	Found^a^ (ng mL^−1^)			
			ELISA	Recovery (%)	Biosensor	Recovery (%)
Skim milk	0	0.10	—	—	0.11	110
		1.0	0.95	95.0	1.04	104
		10	10.2	102	9.91	99.1
		25	24.6	98.4	25.7	103
Milk powder	0	0.10	—	—	0.094	94
		1.0	0.96	96.0	1.06	106
		10	9.82	98.2	10.1	101
		25	25.5	102	24.9	95.6

a Each data point present an average of five independent measurements.

## 4. Conclusions

In summary, by combining target-induced release strategy and HCR signal amplified the flower like nucleic acid nanostructure, we developed an electrochemical sensor for SEB detection with ultrasensitive and specific. RuHex is utilized to amplified signal response. A wide linear range of 0.005–100 ng mL^−1^ and a low detection limit of 3 pg mL^−1^ were obtained for SEB detection. Moreover, the designed sensing technology had a successful application to monitoring SEB in real samples (the skim milk and milk power) and received a good recovery. Therefore, this assay perhaps becomes a valuable tool for determination of SEB in real samples.

## Conflicts of interest

There are no conflicts to declare.

## Supplementary Material

RA-009-C9RA08869E-s001
