# Supplementary material for: Ultrasensitive detection of Staphylococcal enterotoxin B in milk based on target-triggered assembly of the flower like nucleic acid nanostructure
Source: RSC Adv. 2019 Dec 20;9(72):42423–9. doi: 10.1039/c9ra08869e (PMC9076600; doi:10.1039/c9ra08869e)
Supplement: RA-009-C9RA08869E-s001 [file RA-009-C9RA08869E-s001.pdf]

Electronic Supplementary Material (ESI) for RSC Advances.
This journal is © The Royal Society of Chemistry 2019

# Electronic Supporting Information

## Ultrasensitive detection of *Staphylococcal* enterotoxin B in milk based on target-triggered assembly of the flower like nucleic acid nanostructure

Xiaohui Xiong[a,#], Yun Luo[a,#], Yichen Lu[a], Xiong Xiong[a], Yi Li[a], Yuanjian Liu*[a], Lixia Lu*[a]

[a]*Coll Food Sci & Light Ind, Nanjing Tech University, Nanjing 211816, China*

**Corresponding author. Tel.: 86-25-58139432; Fax: 86-25-58139527*

[#]*These authors contributed equally to this work.*

*E-mail address: lucias_cumt@163.com; llxhn66@126.com*

**Table S1**

The sequences for oligonucleotides employed for this work.

| Aauxiliary oligos | Sequence (5' to 3') |
|---|---|
| CpDNA | GATGGCTCTAACTCTCCTCTTTTTT-SH |
| Trigger DNA | AGAGGAGAGTTAGAGCCATCATCATCAGCCGAGAG |
| MB1 | CGAGAGGGGTAGGGCGCCCTCTCGGCTGATGAT |
| MB2 | GGGTAGGGCATCATCTAAGCCCTACCCCTCTCG |
| MB3 | GGCATCATCAGCCGAGAGGATGATGATGCCCTACCC |
| SEB Aptamer | GGTATTGAGGGTCGCATCCACTGGTCGTTGTCTGTTGTCTGTTATGTTGTTTCGTGATGGCTCTAACTCTCCTCT |
